# Development of an AI-Based Suicide Ideation Prediction Model for People with Disabilities

**DOI:** 10.3390/life14111372

**Published:** 2024-10-25

**Authors:** Jimin Han

**Affiliations:** Department of Public Health, Korea University College of Medicine, Seoul 02841, Republic of Korea; 162hcg06@gmail.com

**Keywords:** suicide ideation, artificial intelligence, people with disabilities, prediction model

## Abstract

South Korea has one of the highest suicide rates among countries in the Organisation for Economic Co-Operation and Development, and the suicide rate among people with disabilities is more than twice that of the general population. This study aimed to develop an artificial intelligence-based suicide ideation prediction model for people with disabilities in order to provide a proactive approach for managing high-risk groups and offer evidence for establishing suicide prevention policies. The support vector machine, adaptive boost (AdaBoost), and bidirectional long short-term memory (Bi-LSTM) models were used in this study. Data from the Disability and Life Dynamics Panel for 2018–2021 were used. The performance of the models was evaluated based on the accuracy, sensitivity, specificity, and area under the receiver operating characteristic curve (AUC). All the prediction models demonstrated excellent performance, with AUC > 0.80 (0.83–0.87). The best-performing models were AdaBoost (0.87) for accuracy, Bi-LSTM (0.90) for sensitivity, and AdaBoost (0.90) for specificity. This study is the first to develop an artificial intelligence-based suicide ideation prediction model for disabled people and is significant in that it suggests ways to pre-emptively manage groups at high risk for suicide, providing evidence for the establishment of suicide prevention policies.

## 1. Introduction

Suicide is the major cause of death worldwide, with more than 700,000 people dying by suicide yearly [1]. Therefore, reducing the suicide mortality rate by one-third by 2030 is one of the targets and indicators of the United Nations Sustainable Development Goals (UN SDGs) [1,2] and the World Health Organization’s (WHO) Comprehensive Mental Health Action Plan 2013–2030 [1]. In 2020, South Korea recorded the highest suicide rate among OECD countries [3]. In 2021, the suicide rate was 26.0 per 100,000 people, with an average of 36.6 suicides per day and a total of 13,352 suicides annually, with increases of 0.3, 0.4, and 157, respectively, compared with the previous year [4]. Korea’s suicide rate sharply increased during the 1998 financial crisis and then further increased during the credit card crisis in 2003 and the global financial crisis in 2009, reaching a peak in 2011 (31.7 per 100,000 people). It then decreased until 2017 but slightly increased in 2021, compared with the previous year [4]. Suicide was the leading cause of death among individuals in their 10s to 30s in 2021 and the second-leading cause of death in individuals in their 40s and 50s [4]. Therefore, to prevent suicide, Korea established the ‘Basic Plan for Suicide Prevention Measures’ in 2004 and enacted the ‘Law for Suicide Prevention and Creating a Culture of Respect for Life’ in 2011. The central government, local governments, and the private sector have been working together to promote various suicide prevention projects [4].

The number of registered people with disabilities in South Korea has continuously increased over the past 20 years; it reached 2,641,896 in 2023, accounting for 5.1% of the total population [5]. People with disabilities face difficulties leading independent lives because of their disabilities, which often limit their participation in social activities, including employment. This leads to economic difficulties, which is a major risk factor for suicide. Additionally, the continuous reliance on medical institutions imposes a further economic burden, heightening the likelihood of experiencing psychological distress such as depression and anxiety [6]. In the 2021 Mental Health Screening for People with Disabilities, significant percentages of people were suffering from depression, a major risk factor for suicide, with people in their 20s, 30s, 40s, 50s, 60s, and 70s having depression rates of 23.1%, 28.2%, 29.9%, 23.5%, 19.9%, and 14.2%, respectively [7].

Suicide is also a major cause of death in people with disabilities, ranking as the third leading cause of death among those in their 20s and second among those in their 30s as of 2022 [7]. Moreover, the crude death rate for people with disabilities in 2022 was 53.1 per 100,000 people [8], which was more than double the crude death rate of 25.2% in the general population [8]. Therefore, continued efforts at the national level, including legal and institutional reforms, are necessary to prevent suicide in people with disabilities.

The WHO recommends that each country establishes and promotes government-led suicide prevention strategies, as follows: (1) restriction of access to suicide means; (2) interaction with the media for responsible suicide reporting; (3) promotion of adolescents’ social–emotional life skills; and (4) early identification, evaluation, management, and follow-up of individuals affected by suicidal behaviour [1]. Suicide ideation often leads to suicide; hence, it has been used as an important primary indicator for predicting suicide [9] and as evidence for establishing policies for suicide prevention. Therefore, various studies have been conducted to identify the factors that influence suicide ideation among people with disabilities in South Korea. A review of key prior studies showed that many studies did not differentiate between the types of disabilities [9,10,11,12,13,14,15,16,17,18,19], while others focused on specific disabilities, such as kidney disorders [20], mental disorders [21,22], or physical and brain disabilities [6,23,24,25,26,27]. However, most previous studies used the results of a single survey, causing a wide variation in the reported effects of population and sociological factors, disability and health factors, socioeconomic conditions, and psychological and environmental influences on the suicide ideation of people with disabilities. Consequently, the factors influencing suicide ideation among people with disabilities have remained unclear. Therefore, further research is needed to identify the determinants of suicide ideation among individuals with disabilities.

Recently, there has been an increasing trend in the use of big data and artificial intelligence (AI) in suicide prevention strategies. In the United States, the Durkheim Project was implemented to prevent suicide among specific groups (such as veterans) by integrating public data platforms, such as social media, with healthcare databases and using machine learning to monitor real-time linguistic and behavioural patterns statistically associated with suicide [28,29]. In New York, the Office of Mental Health has utilised various databases, including the Psychiatric Services and Clinical Knowledge Enhancement System (PSYCKES), to develop data-driven suicide prevention strategies [30,31]. Additionally, Australia’s Black Dog Institute has launched the LifeSpan Project, which uses big data to identify regions with high suicide rates and limit access to means of suicide by establishing suicide prevention infrastructure in those areas [28]. Such attempts have continued in Korea, and in February 2023, using a suicide prevention system based on AI technology, Tongyeong saved a woman in her 20s who tried to die by suicide [32]. Furthermore, in May 2023, the Presidential Suicide Crisis Overcome Special Committee emphasised the need for a scientific and precise suicide prevention policy by developing a “suicide prediction model” that could identify high-risk groups early, including the potential for integrating AI technologies [33].

Therefore, this study aimed to develop an AI-based suicide ideation prediction model using a Disability and Life Dynamics Panel that is representative of people with disabilities in South Korea. This study sought to propose proactive management strategies for high-risk groups and provide evidence for the development of suicide prevention policies for people with disabilities. For this purpose, support vector machine (SVM) and adaptive boost (AdaBoost) of machine learning, and bidirectional long short-term memory (Bi-LSTM) of deep learning were selected as study models.

## 2. Materials and Methods

### 2.1. Study Population and Ethical Considerations

This study selected the Disability and Life Dynamics Panel (Approval No. 438001) [34] as the analysis data. These include individuals with disabilities and their household members who registered their disabilities with the Ministry of Health and Welfare between 1 January 2015 and 31 December 2017, in accordance with Article 32 of the Act on Welfare of Persons with Disabilities. The Life Dynamics Panel was launched in 2018 to provide foundational data for formulating welfare policies for people with disabilities by understanding the process of disability acceptance and the changes experienced in social relationships. The survey covered the following areas: (1) acceptance of and changes in disability; (2) health and medical care; (3) independence; and (4) social participation [34].

The data for this study were obtained from the Korea Disabled People’s Development Institute, and 19,141 responses from the 2018 (1st wave) to 2021 (4th wave) surveys, which included answers to the dependent variable, suicide ideation, were used.

Ethical approval was not required because this study utilised existing survey data. Therefore, an exemption from review was granted by the Korea University Institutional Review Board (KUIRB-2023-0413-01) on 1 December 2023.

### 2.2. Variables and Categories

The dependent variable was suicide ideation. Through an analysis of 13 previous studies on factors influencing suicide ideation among people with disabilities [6,9,10,11,12,13,14,16,20,21,23,24,25], 39 independent variables were selected (Table 1).

### 2.3. Statistical Analysis

AI encompasses machine and deep learning and is often defined as research aimed at automating tasks that humans perform intelligently [42]. Machine learning primarily focuses on predicting the outcomes for new data by learning from existing data [43], whereas deep learning, a subset of machine learning, excels at learning meaningful representations through sequential layers, making it particularly effective at learning representations from data [42].

AI encompasses different learning methods, each with differing learning effects based on data suitability and on the algorithm being used. As such, this study attempted to compare the prediction results of the models based on three algorithms. For this study, the AI models selected were SVM, AdaBoost from the field of machine learning, and Bi-LSTM from deep learning. Data pre-processing and analysis were conducted using Python version 3.12.4. During the pre-processing stage, continuous variables were standardised by adjusting their mean and variance to zero and one, respectively [44], whereas target encoding was applied to categorical variables by replacing individual categories with their mean values [45]. The dataset was split into 99.5% for training data and 0.5% for testing data.

Given that only 8.48% (*n* = 1623) of the participants (*n* = 19,141) reported experiencing suicide ideation, there was a class imbalance in the data. To address this imbalance, oversampling was performed on the training data using the synthetic minority oversampling technique, nominally continuous (SMOTE-NC), which is suitable for datasets containing both categorical and continuous variables [46]. For model interpretation, the permutation feature importance (PFI) approach was applied, which measures the change in model performance when the values of the variables are shuffled or permuted [47]. The performance of the models was evaluated using accuracy, sensitivity, specificity, and the area under the receiver operating characteristic (ROC) curve (AUC) [48].

#### 2.3.1. Confusion Matrix

The definition of the four parameters used to evaluate model performance can be explained using a confusion matrix (Table 2) [48].

■ True positive: A case in which a model correctly predicts a positive value when the actual value is positive.
■ True negative: A case in which a model correctly predicts a negative when the actual value is negative.
■ False positive: A case in which a model incorrectly predicts a positive value when the actual value is negative.
■ False negative: A case in which a model incorrectly predicts a negative value when the actual value is positive.

#### 2.3.2. Accuracy

Accuracy is an indicator that represents the proportion of correct predictions from the total data [48]. Accuracy refers to the accuracy of the model’s predictions for the entire dataset, including positive and negative instances of suicide ideation.

$$\mathrm{Accuracy}=\frac{\mathrm{TP}+\mathrm{TN}}{\mathrm{TP}+\mathrm{FP}+\mathrm{TN}+\mathrm{FN}}$$

#### 2.3.3. Sensitivity (True Positive Rate or Recall)

Sensitivity is an indicator that represents the proportion of actual positive data that the model correctly predicts as positive [48]. In this study, sensitivity refers to the proportion of individuals with suicide ideation correctly identified by the model.

$$\mathrm{Sensitivity}=\frac{\mathrm{TP}}{\mathrm{TP}+\mathrm{FN}}$$

#### 2.3.4. Specificity (True Negative Rate)

Specificity is an indicator representing the proportion of actual negative data that the model correctly predicts as negative [48]. Specificity refers to the proportion of individuals without suicide ideation that the model correctly identified as not having suicide ideation.

$$\mathrm{Specificity}=\frac{\mathrm{TN}}{\mathrm{TN}+\mathrm{FP}}$$

#### 2.3.5. AUC

The ROC curve is a graph that plots sensitivity on the y-axis and 1-specificity on the x-axis, illustrating the trade-off between true positive and false positive rates at various threshold levels [48]. The AUC represents the area under this curve and is used to evaluate the overall classification performance of the model [48].

## 3. Results

### 3.1. Descriptive Statistics

The results of the descriptive statistics analysis of the participants of this study are shown in Table 3.

**Figure 1:**
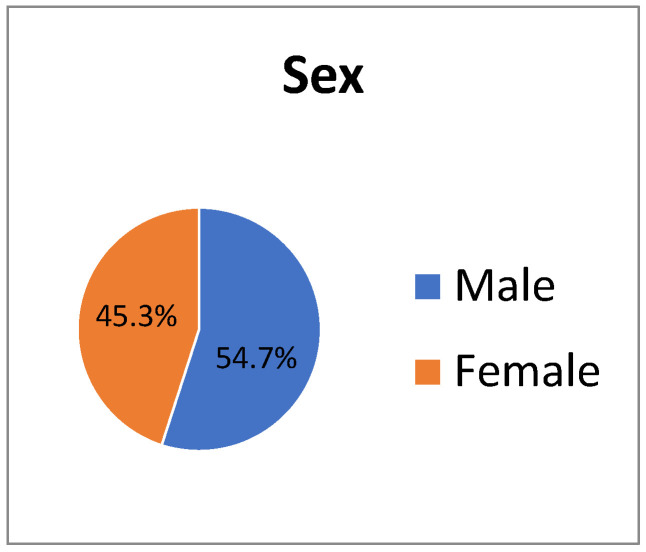
Distribution of sex.

**Figure 2:**
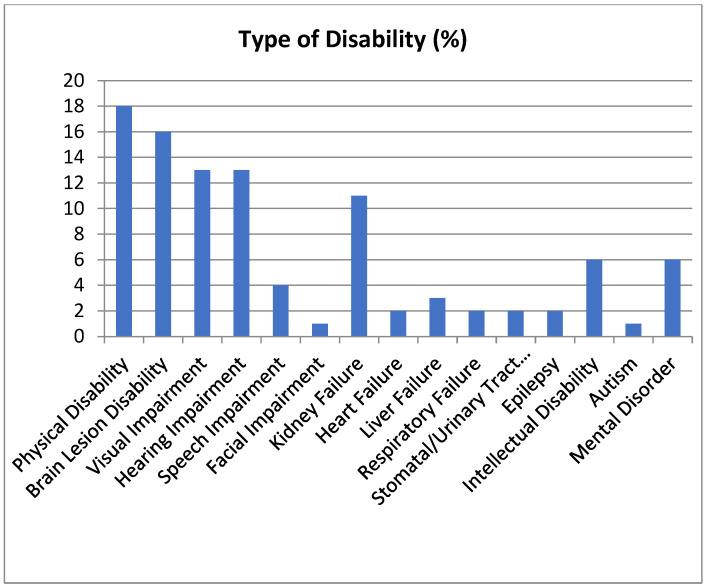
Distribution of type of disability.

The study participants (n = 19,141) comprised 54.7% male and 45.3% female (Figure 1). The age distribution was as follows: 4.3% in their 10s, 6.2% in their 20s, 5.9% in their 30s, 11.6% in their 40s, 27.6% in their 50s, 35.4% in their 60s, and 9% in their 70s, with the largest group being those in their 60s. Regarding marital status, 23%were unmarried, 52% married, and 25% divorced/separated/bereaved. In terms of disability type, 17.4% had physical disability, 15.6% had brain lesion disability, 13.3% had visual impairment, 12.5% had hearing impairment, 4.3% had speech impairment, 0.5% had facial disability, 10.6% had kidney failure, 2.1% had heart failure, 3.6% had liver failure, 2.7% had respiratory failure, 2.5% had stomatal urinary tract disorder, 2.1% had epilepsy, 5.8% had intellectual disability, 1% had autism, and 6%had a mental disorder (Figure 2). Physical disabilities were the most prevalent type.

### 3.2. SVM

SVM is a traditional machine learning technique that can be applied to both classification and regression problems, offering high generalisation performance [49,50]. It is frequently used because of its simplicity, computational efficiency, and ability to train with small datasets [51,52,53]. To solve classification problems, such as the one in this study, SVM aims to find an optimal boundary between two different categories, where the optimal boundary is a line or surface that separates the training data into regions corresponding to each category [42].

For the SVM model, the hyperparameters selected were cost and linear kernel. Through optimisation, we found that when the cost was set to 8.24, the SVM achieved an accuracy of 0.82, sensitivity of 0.66, specificity of 0.83, and AUC of 0.83. Additionally, after applying the PFI approach, the most important variables were employment type, employment status, job satisfaction, age, depression, marital satisfaction, experience using social welfare facilities, leisure activity satisfaction, satisfaction with the use of social welfare facilities, and severity of disability (Table 4).

### 3.3. AdaBoost

AdaBoost, an ensemble learning technique, is a boosting method first designed to improve binary classification performance [48,54,55]. AdaBoost learns from weak decision trees, known as stumps, and combines them to form an ensemble [55]. Weak classifiers perform slightly better than random estimates, and AdaBoost amplifies their functionality to create a strong classifier [48]. AdaBoost can be applied to various classification problems [55], and although it is not specifically designed for datasets with class imbalance, it helps to address such problems by assigning different weights to each class [48].

The hyperparameters of AdaBoost were selected as N_estimators and learning rate. After optimisation, the results were as follows: N_estimators = 133, learning rate = 0.38, accuracy = 0.87, sensitivity = 0.62, specificity = 0.90, and AUC = 0.87. Additionally, after applying the PFI approach, the most important variables were age, average number of exercise days per week, smoking status, satisfaction with residential environment, area size, number of household members, severity of disability, satisfaction with welfare services, duration of disability, and leisure activity satisfaction (Table 5).

### 3.4. Bi-LSTM

LSTM has been proposed to address the shortcomings of RNNs [42,56] and solve the vanishing gradient problem of RNNs by injecting past information at later stages [42]. LSTM networks are designed to transfer important information across multiple future steps and typically include three gates: input, output, and forget [57]. Bi-LSTM enhances the performance of traditional LSTM by adding a backward memory block (or cell) to the LSTM memory block, thereby facilitating end-to-end learning in a deep learning model [58].

The hyperparameters for Bi-LSTM were batch size, hidden size, and iterations (Iter). After optimisation, the results were as follows: batch size = 128, hidden size = 5, iteration = 40, accuracy = 0.67, sensitivity = 0.90, specificity = 0.65, and AUC = 0.87. Additionally, the PFI approach revealed that the most important variables were age, job satisfaction, marital satisfaction, leisure activity satisfaction, severity of disability, disability-related daily life restrictions, satisfaction with welfare services, area size, number of meals a day, and satisfaction with the use of social welfare facilities(Table 6).

## 4. Discussion

This study developed an AI-based suicide ideation prediction model for people with disabilities to provide strategies for the proactive management of high-risk groups by national and medical institutions. In addition, this study selected three models (SVM, AdaBoost, and Bi-LSTM) based on different algorithms to compare their prediction results. Our results showed that the AUCs for the models were 0.83 (SVM), 0.87 (AdaBoost), and 0.87 (Bi-LSTM) for SVM, AdaBoost, and Bi-LSTM, respectively. Notably, all the AUC values exceeded 0.80, indicating excellent performance. Generally, an AUC > 0.80 is considered outstanding [59,60]. Considering the performance of each indicator, the model with the highest accuracy, which represents the accuracy of the results predicted by the model in the overall data, was AdaBoost (0.87). Bi-LSTM had the highest sensitivity (0.90), which represents the rate of correctly predicting suicide ideation. AdaBoost also had the highest specificity (0.90), which represents the percentage of those who did not have suicide ideation. If sensitivity, the rate of correctly identifying true positives, is the most important criterion, Bi-LSTM would be the most appropriate model. In addition, the prediction model developed in this study can be used to identify high-risk groups for suicide ideation and to develop a system that can intensively manage these groups. The variables influencing suicide ideation for disabled people, as presented in this study, can be used to establish suicide prevention policies for people with disabilities or treatment methods aimed at improving their mental health.

This study used the PFI approach to identify the importance of the variables for each model. The top ten variables common across all three models included age, severity of disability, and leisure activity satisfaction, which are closely related to physical functioning. In addition, the variables commonly included in the top ten in two of the three models were marital satisfaction, satisfaction with welfare services, area size, job satisfaction, and satisfaction with the use of social welfare facilities, which are closely related to daily life. This means that in the three algorithms, these variables commonly acted as important variables to predict the suicide ideation of people with disabilities. These findings differ from those of previous studies that identified depression and pessimistic self-perception as major risk factors for suicide or suicide ideation [61,62,63,64,65], likely because of differences in the study population or methodology.

Our review of prior studies revealed only four studies that included people with disabilities as part of their research population on AI-based suicide ideation prediction [59,61,66,67], highlighting the limitations of previous research in applying their findings to the disabled population. As people with disabilities have a higher suicide rate than the general population, the need to identify the difference between the disabled and the general population has become increasingly important. However, this distinction has not yet been achieved. Therefore, this study is the first to develop a model for predicting suicide ideation for people with disabilities. It presents the risk factors for suicide ideation among people with disabilities that are distinct from those of the general population, which can be used as basic data for establishing suicide prevention policies for people with disabilities.

Suicide is a global issue. Taiwan enacted the Suicide Prevention and Elimination Act in 2019 owing to the increasing suicide rate [68], while Germany revised the Children and Youth Support Act enacted in 1991 to the Child and Youth Reinforcement Act in 2021 [69]. In addition, the French Minister of Education announced mental health promotion measures in May 2023 to provide education to detect student vulnerability and urge all middle and high school students to include suicide prevention national phone numbers in their communication letters [69]. To date, countries have made efforts to lower suicide rates. In addition, to reduce the increasing suicide rate, Korea has revised its laws to prevent suicide and create a culture of respect for life, implementing suicide prevention projects that manage the use of carbon monoxide, pesticides, and bridges, which are used as suicide means [70]. Additionally, in April, the Korea Suicide Prevention Association issued an emergency statement calling for counter measures against suicide [71], which showed the urgency to develop innovative measures to reduce the suicide rate in Korea.

The current study used machine learning models, including SVM and AdaBoost, as well as the deep learning model Bi-LSTM, and analysed 19,141 data points from the 2018–2021 Disability and Life Dynamics Panel. Notably, machine and deep learning models generally perform better with larger datasets; thus, the 19,141 data points used in this study may be insufficient. Furthermore, this study selected 39 independent variables based on previous research [6,9,10,11,12,13,14,16,20,21,23,24,25]; however, there may be other important variables that can predict suicide ideation among people with disabilities. Therefore, future research on this topic may consider exploiting more variables, data, and additional models other than SVM, AdaBoost, and Bi-LSTM. In addition, because this study was conducted based on Korean disabled data, the results of this study may not be applicable to countries other than Korea. Furthermore, this study did not present the results based on the type of disability due to insufficient data for each type of disability. Therefore, future studies may consider presenting results based on the type of disability. This study utilised the results of a previous survey; hence, it is difficult to rule out the possibility that some information was omitted. Additionally, this study did not account for participants’ internal pain levels and whether they were receiving treatment for mental illness.

## 5. Conclusions

This study is the first to develop an AI-based suicide ideation prediction model for people with disabilities, using 19,141 data points from the ‘Disability and Life Dynamics Panel’ survey, which is representative of the disabled population in South Korea. The results showed that all three models (SVM, AdaBoost, and Bi-LSTM) achieved an AUC > 0.80. Additionally, this study employed the PFI approach to calculate the importance of variables in each model, identifying the factors influencing suicide ideation among people with disabilities that differ from those affecting the general population. The findings of this study provide significant evidence that can be used for the proactive management of high-risk groups and the formulation of suicide prevention policies for people with disabilities.

## Figures and Tables

**Table 1:** Variables and categories.

No.	Variables	Category
1	Age	1: Under 10 years old 2: 10s 3: 20s 4: 30s 5: 40s 6: 50s 7: 60s 8: 70s or older
2	Sex	1: Male 2: Female
3	Area size	1: Town 2: Small–medium city 3: Large city
4	Marital status	1: Single 2: Married 3: Divorce/separation/bereavement
5	Number of household members	1: Single-person households 2: Two-person households 3: Three-person households 4: More than four-person households
6	Education level	1: Unschooled 2: Preschool 3: Elementary school 4: Middle school 5: High school 6: Junior college 7: Bachelor 8: Master 9: Doctor
7	Severity of disability	1: Mild (Level 4 to 6 based on the level of disability in Korea) 2: Severe (Level 1 to Level 3 based on the level of disability in Korea)
8	Duration of disability	1: Less than 5 years 2: 5 to 9 years 3: 10 to 19 years 4: 20 years or more
9	Type of disability	1: Physical disability 2: Brain lesion disability 3: Visual impairment 4: Hearing impairment 5: Speech impairment 6: Facial impairment 7: Kidney failure 8: Heart failure 9: Liver failure 10: Respiratory failure 11: Stomatal urinary tract disorder 12: Epilepsy 13: Intellectual disability 14: Autism 15: Mental disorder
10	Cause of disability	1: Innate cause 2: Acquired cause
11	Multiple disabilities	1: Yes 2: None
12	Number of chronic diseases	1: No chronic diseases 2: More than 1 chronic disease, less than 2 chronic diseases 3: More than 3 chronic diseases
13	Disability acceptance	1: Not at all (1 point) 2: That is hardly the case (2 points) 3: That is what it is (3 points) 4: Very much so (4 points)
14	Depression	1: Extremely rare (0 points) 2: Occasionally (1 point) 3: Often (2 points) 4. Most of the time (3 points)
15	Self-esteem	1: Not at all (1 point) 2: That is not true (2 points) 3: Yes (3 points) 4: That is always the case (4 points)
16	Satisfaction with family relationships	1: Not at all (1 point) 2: That is not true (2 points) 3: Yes (3 points) 4: That is always the case (4 points)
17	Family strengths	1: That is not true (1 point) 2: Sometimes that is not the case (2 points) 3: Sometimes that is the case (3 points) 4: That is always the case (4 points)
18	A subjective health condition	1: Very bad 2: Bad 3: Good 4: Very good
19	Average number of exercise days per week	1: No exercise 2: One 3: Two 4: Three 5: Four 6: Five 7: Six 8: Seven
20	Number of meals a day	1: Less than one 2: One 3: Two 4: Three 5: Four or more
21	Smoking status	1: Smoking 2: Smoking e-cigarettes 3: Smoking in the past 6 months but not smoking previously 4: Never smoked 5: Smoking both cigarettes and e-cigarettes
22	Drinking status	1: Never drank alcohol 2: Drank previously but not in the past 6 months 3: Drinks alcohol
23	Presence of a caregiver for daily life assistance	1: Yes 2: None
24	Disability- related daily life restrictions	1: Not at all (1 point) 2: Almost none (2 points) 3: Fairly present (3 points) 4: Very much (4 points)
25	Experience in welfare services related to persons with disabilities	1: Yes 2: None
26	Experience in using social welfare facilities	1: Yes 2: None
27	Satisfaction with welfare services	1: Very dissatisfied 2: Very unsatisfied 3: Satisfied 4: Very satisfied
28	Satisfaction with the use of social welfare facilities	1: Not satisfied at all 2: Not satisfied at all 3: Satisfied 4: Very satisfied
29	Experiences of bullying or violence	1: Yes 2: None
30	Leisure activity satisfaction	1: Not satisfied at all 2: Not satisfied at all 3: Satisfied 4: Very satisfied
31	Employment status	1: Employed 2: Unemployed
32	Employment type	1: Regular worker 2: Temporary worker 3: Daily worker 4: Self-employed person 5: Self-employed but does not employ employees 6: Not being paid and helping the family business
33	Financial preparation for old age	1: I prepared it 2: I did not prepare it
34	Current income satisfaction	The closer to 10 than 1, the higher the satisfaction level
35	Satisfaction with residential environment	The closer to 10 than 1, the higher the satisfaction level
36	Job satisfaction	The closer to 10 than 1, the higher the satisfaction level
37	Marital satisfaction	The closer to 10 than 1, the higher the satisfaction level
38	Social relationship satisfaction	The closer to 10 than 1, the higher the satisfaction level
39	Life satisfaction	The closer to 10 than 1, the higher the satisfaction level

Note: The disability period refers to a period from the diagnosis of the disability to the time of investigation [34]. Disability acceptance means acknowledging life changes caused by disability and finding another life value [35,36]. Depression, unlike general mood swings, refers to a long-lasting depressive mood, absence of pleasure, or loss of interest, which can affect all aspects of life, including family, friends, and community [37]. Self-esteem is a self-evaluation of one’s own worth, and high self-esteem means evaluating oneself as a valuable being [34,38]. Family strength refers to the degree of health of a family by having an optimistic and positive attitude toward life from a strong family perspective [39,40]. Social welfare facilities are facilities established for social welfare programs defined in Article 2 of the Social Welfare Services Act (SOCIAL WELFARE SERVICES ACT), and social welfare programs are projects aimed at operating or supporting various welfare programs and related volunteer activities and welfare facilities [34,41].

**Table 2:** Confusion matrix.

	Positive	Negative
Positive	TP	FN
Negative	FP	TN

**Table 3:** Results of descriptive statistics.

Variables		Have Suicidal Ideation	No Suicidal Ideation	Total
		N (%)	N (%)	N (%)
Sex	Male	859 (4.5)	9618 (50.2)	10,477 (54.7)
	Female	764 (4)	7900 (41.3)	8664 (45.3)
Age	10s	38 (0.2)	784 (4.1)	822 (4.3)
	20s	98 (0.5)	1095 (5.7)	1193 (6.2)
	30s	108 (0.6)	1019 (5.3)	1127 (5.9)
	40s	238 (1.2)	1980 (10.3)	2218 (11.6)
	50s	517 (2.7)	4768 (24.9)	5285 (27.6)
	60s	509 (2.7)	6258 (32.7)	6767 (35.4)
	70s or older	115 (0.6)	1614 (8.4)	1729 (9)
Marital status	Single	373 (1.9)	4023 (21)	4396 (23)
	Married	725 (3.8)	9231 (48.2)	9956 (52)
	Divorce/separation/bereavement	525 (2.7)	4264 (22.3)	4789 (25)
Type of disability	Physical disability	290 (1.5)	3046 (15.9)	3336 (17.4)
	Brain lesiondisability	309 (1.6)	2673 (14)	2982 (15.6)
	Visual impairment	204 (1.1)	2343 (12.2)	2547 (13.3)
	Hearing impairment	125 (0.7)	2266 (11.8)	2391 (12.5)
	Speech impairment	56 (0.3)	761 (4)	817 (4.3)
	Facial impairment	22 (0.1)	73 (0.4)	95 (0.5)
	Kidney failure	178 (0.9)	1845 (9.6)	2023 (10.6)
	Heart failure	19 (0.1)	384 (2)	403 (2.1)
	Liver failure	45 (0.2)	640 (3.3)	685 (3.6)
	Respiratory failure	55 (0.3)	466 (2.4)	521 (2.7)
	Stomatalurinary tract disorder	45 (0.2)	428 (2.2)	473 (2.5)
	Epilepsy	67 (0.4)	344 (1.8)	411 (2.1)
	Intellectual disability	56 (0.3)	1053 (5.5)	1109 (5.8)
	Autism	3 (0)	189 (1)	192 (1)
	Mental disorder	149 (0.8)	1007 (5.3)	1156 (6)

**Table 4:** Permutation importance of SVM.

No.	Variables	Feature Importance	No.	Variables	Feature Importance
1	Employment type	0.0919 ± 0.0089	21	Experience in welfare services related to persons with disabilities	0.0004 ± 0.0039
2	Employment status	0.0638 ± 0.0072	22	Disability-related daily life restrictions	0.0003 ± 0.0016
3	Job satisfaction	0.0419 ± 0.0051	23	Financial preparation for old age	0.0001 ± 0.0003
4	Age	0.0190 ± 0.0033	24	Presence of a caregiver for daily life assistance	0.0001 ± 0.0029
5	Depression	0.0181 ± 0.0090	25	Number of meals a day	0.0000 ± 0.0012
6	Marital satisfaction	0.0091 ± 0.0058	26	Type of disability	−0.0004 ± 0.0032
7	Experience in using social welfare facilities	0.0069 ± 0.0013	27	Number of household members	−0.0005 ± 0.0030
8	Leisure activity satisfaction	0.0060 ± 0.0039	28	Sex	−0.0010 ± 0.0014
9	Satisfaction with the use of social welfare facilities	0.0045 ± 0.0018	29	Satisfaction with family relationships	−0.0015 ± 0.0024
10	Severity of disability	0.0036 ± 0.0019	30	Disability acceptance	−0.0018 ± 0.0018
11	Education level	0.0023 ± 0.0051	31	Current income satisfaction	−0.0022 ± 0.0042
12	Multiple disabilities	0.0022 ± 0.0019	32	Social relationship satisfaction	−0.0022 ± 0.0018
13	Area size	0.0022 ± 0.0018	33	Cause of disability	−0.0023 ± 0.0028
14	Drinking status	0.0021 ± 0.0030	34	Family strengths	−0.0024 ± 0.0045
15	Satisfaction with welfare services	0.0018 ± 0.0031	35	Number of chronic diseases	−0.0032 ± 0.0021
16	Duration of disability	0.0016 ± 0.0035	36	Life satisfaction	−0.0041 ± 0.0019
17	Smoking status	0.0016 ± 0.0045	37	A subjective health condition	−0.0053 ± 0.0042
18	Marital status	0.0013 ± 0.0021	38	Satisfaction with residential environment	−0.0063 ± 0.0032
19	Experiences of bullying or violence	0.0008 ± 0.0016	39	Self-esteem	−0.0070 ± 0.0019
20	Average number of exercise days per week	0.0007 ± 0.0017			

**Table 5:** Permutation importance of AdaBoost.

No.	Variables	Feature Importance	No.	Variables	Feature Importance
1	Age	0.0046 ± 0.0051	21	Experience in welfare services related to persons with disabilities	−0.0005 ± 0.0017
2	Average number of exercise days per week	0.0016 ± 0.0017	22	Job satisfaction	−0.0006 ± 0.0026
3	Smoking status	0.0015 ± 0.0010	23	Type of disability	−0.0007 ± 0.0031
4	Satisfaction with residential environment	0.0014 ± 0.0018	24	Cause of disability	−0.0007 ± 0.0011
5	Area size	0.0007 ± 0.0009	25	Social relationship satisfaction	−0.0008 ± 0.0026
6	Number of household members	0.0005 ± 0.0020	26	Number of chronic diseases	−0.0008 ± 0.0015
7	Severity of disability	0.0005 ± 0.0007	27	Number of meals a day	−0.0008 ± 0.0011
8	Satisfaction with welfare services	0.0004 ± 0.0012	28	Marital status	−0.0009 ± 0.0005
9	Duration of disability	0.0003 ± 0.0019	29	A subjective health condition	−0.0014 ± 0.0014
10	Leisure activity satisfaction	0.0003 ± 0.0019	30	Satisfaction with family relationships	−0.0016 ± 0.0013
11	Life satisfaction	0.0002 ± 0.0020	31	Drinking status	−0.0016 ± 0.0009
12	Satisfaction with the use of social welfare facilities	0.0001 ± 0.0003	32	Disability-related daily life restrictions	−0.0019 ± 0.0028
13	Experiences of bullying or violence	0.0001 ± 0.0016	33	Current income satisfaction	−0.0021 ± 0.0009
14	Depression	0.0001 ± 0.0035	34	Multiple disabilities	−0.0023 ± 0.0007
15	Employment status	0 ± 0.0000	35	Experience in using social welfare facilities	−0.0026 ± 0.0011
16	Disability acceptance	−0.0001 ± 0.0018	36	Employment type	−0.0033 ± 0.0012
17	Family strengths	−0.0002 ± 0.0015	37	Financial preparation for old age	−0.0040 ± 0.0008
18	Self-esteem	−0.0003 ± 0.0014	38	Presence of a caregiver for daily life assistance	−0.0048 ± 0.0009
19	Education level	−0.0004 ± 0.0031	39	Sex	−0.0050 ± 0.0015
20	Marital satisfaction	−0.0004 ± 0.0018			

**Table 6:** Permutation importance of Bi-LSTM.

No.	Variables	Feature Importance	No.	Variables	Feature Importance
1	Age	0.0043 ± 0.0004	21	Experiences of bullying or violence	−0.0000 ± 0.0000
2	Job satisfaction	0.0040 ± 0.0003	22	Cause of disability	−0.0000 ± 0.0000
3	Marital satisfaction	0.0037 ± 0.0004	23	Presence of a caregiver for daily life assistance	−0.0000 ± 0.0000
4	Leisure activity satisfaction	0.0026 ± 0.0003	24	Drinking status	−0.0000 ± 0.0000
5	Severity of disability	0.0016 ± 0.0002	25	Education level	−0.0000 ± 0.0000
6	Disability-related daily life restrictions	0.0014 ± 0.0002	26	Employment type	−0.0001 ± 0.0000
7	Satisfaction with welfare services	0.0013 ± 0.0007	27	Type of disability	−0.0002 ± 0.0000
8	Area size	0.0012 ± 0.0003	28	Satisfaction with residential environment	−0.0002 ± 0.0002
9	Number of meals a day	0.0004 ± 0.0004	29	Employment status	−0.0006 ± 0.0000
10	Satisfaction with the use of social welfare facilities	0.0001 ± 0.0002	30	Number of chronic diseases	−0.0008 ± 0.0005
11	Number of household members	0.0001 ± 0.0002	31	Disability acceptance	−0.0019 ± 0.0003
12	Duration of disability	0.0001 ± 0.0002	32	Family strengths	−0.0021 ± 0.0003
13	Experience in welfare services related to persons with disabilities	0.0001 ± 0.0000	33	Satisfaction with family relationships	−0.0029 ± 0.0004
14	Financial preparation for old age	0.0001 ± 0.0000	34	Current income satisfaction	−0.0033 ± 0.0004
15	Smoking status	0.0000 ± 0.0000	35	A subjective health condition	−0.0037 ± 0.0005
16	Average number of exercise days per week	0.0000 ± 0.0002	36	depression	−0.0037 ± 0.0012
17	Marital status	0.0000 ± 0.0000	37	Self-esteem	−0.0037 ± 0.0003
18	Sex	0.0000 ± 0.0000	38	Life satisfaction	−0.0042 ± 0.0004
19	Experience in using social welfare facilities	0.0000 ± 0.0000	39	Social relationship satisfaction	−0.0053 ± 0.0005
20	Multiple disabilities	0.0000 ± 0.0000			

## Data Availability

No new data were created or analyzed in this study.
